# Sensory Response Patterns in Nonverbal Children with ASD

**DOI:** 10.1155/2013/436286

**Published:** 2013-07-15

**Authors:** Elena Patten, Karla K. Ausderau, Linda R. Watson, Grace T. Baranek

**Affiliations:** ^1^Department of Communication Sciences and Disorders, The University of North Carolina, Greensboro, 300 Ferguson Building, Greensboro, NC 27412, USA; ^2^The University of Wisconsin-Madison, 3195 Medical Sciences Center, 1300 University Avenue, Madison, WI 53706, USA; ^3^Division of Speech & Hearing Sciences, The University of North Carolina at Chapel Hill, CB No. 7190 Chapel Hill, NC 27599, USA; ^4^Division of Occupational Science & Occupational Therapy, The University of North Carolina at Chapel Hill, CB No. 7122 Chapel Hill, NC 27599, USA

## Abstract

We sought to examine concurrent and longitudinal associations between sensory response patterns (i.e., hyperresponsiveness, hyporesponsiveness, and sensory seeking) and verbal status of young children with autism spectrum disorder (ASD) as a potential factor influencing the development of verbal communication. Seventy-nine children with ASD (verbal, *n* = 29; nonverbal, *n* = 50) were assessed using cross-sectional analyses (Study 1), and 14 children with ASD (verbal, *n* = 6; nonverbal, *n* = 8) were assessed using prospective longitudinal analyses (Study 2). Data were collected regarding sensory response patterns and verbal ability. Hyporesponsiveness and sensory seeking behaviors were associated with verbal status in both cross-sectional and longitudinal analyses; nonverbal children were more likely to demonstrate higher hyporesponsive and sensory seeking patterns. Hyperresponsiveness did not significantly differ between verbal and nonverbal groups in either design. Sensory hyporesponsiveness and seeking behaviors may be important factors hindering the development of functional verbal communication in children with ASD. Unusual sensory responsiveness can often be observed before the onset of speech and may yield important prognostic capabilities as well as inform early interventions targeting verbal communication or alternative communication options in young children with ASD.

## 1. Introduction

Approximately 25–50% of children with autism spectrum disorder (ASD) do not develop functional verbal communication [1–3], and functional verbal communication rarely develops after age of five in children with ASD [4]. These nonverbal children have significantly poorer outcomes compared to children with ASD who do develop functional verbal communication; development of speech prior to this age predicts educational achievement, employment, the ability to live independently, and social relationships [5, 6]. Variability exists regarding terminology defining expressive communication in children with ASD, particularly regarding the term “functional verbal communication” [7]. For purposes of this paper, we conceptualize functional verbal communication based on discussions by Baghdadli et al. [1] and Tager-Flusberg et al. [7] as *spoken language that is spontaneous and meaningful and includes multiword utterances*. 

Although nonverbal children with ASD comprise a substantial portion of the population and outcomes are poor for them, limited research elucidating potential explanatory factors for the failure of some children with ASD to acquire meaningful expressive language or examining treatments that may improve long-term outcomes is available for individuals who remain nonverbal into the school-age years. To address this issue, the National Institutes of Health (NIH) conducted a workshop to discuss the state of the science and to generate research priorities regarding nonverbal children with ASD [8]. One research priority identified by participants in this workshop was to identify mechanisms underlying the lack of development of functional verbal communication that occurs in some children with ASD. If auditory and visual information provided by communication models are not adequately processed, verbal communication may not develop normally [9]. Abnormal sensory processing of environmental stimuli may be one mechanism underlying both sensory features and impaired development of functional verbal communication in children with autism. Thus, we are interested in evaluating sensory response patterns that are manifest and observable early in life that may be associated with the development of functional verbal communication. 

The ability to accurately process environmental stimuli, particularly early in development, is necessary for typical development of a number of early social and communication skills. Impairments in sensory processing are commonly associated with ASD; a meta-analysis by Ben-Sasson and colleagues [10] suggested that, to some degree, sensory processing disturbances may be universal in ASD. Increased frequency of unusual sensory features in individuals with ASD has been documented through self-reports [11] as well as through numerous empirical studies (e.g., [12–15]). More importantly, severity of sensory features in children with ASD has been found to predict various adaptive and maladaptive behaviors such as communication [9], socialization [16], and stereotyped behaviors [17].

The ability to process and respond to sensory stimuli has been conceptualized in the literature and tested in a variety of ways. Although in natural environments stimuli are multimodal, experimental studies often test single sensory modalities (e.g., vision, audition, and touch) (e.g., [18–20]). Some research teams have shown utility for categorizing responses to sensory stimuli across multiple modalities as reflecting at least three broad patterns or constructs: hyporesponsive, hyperresponsive, and sensory seeking [17, 21–24]. Each sensory response pattern is thought to represent a continuum related to the frequency or intensity with which sensory features characteristic of that pattern are observed. Hyporesponsiveness is characterized by delayed, absent, or more attenuated responses to stimuli than expected (e.g., child requires multiple or intense auditory cues to elicit an orienting response) [17, 21–24]. Hyperresponsiveness is characterized by an exaggerated, often negative, response to stimuli (e.g., child attempts to avoid stimulation, such as covering ears to filter certain sounds, or shows an aversive response to tactile stimuli that would not bother others) [17, 21–24]. Sensory seeking encompasses behaviors that appear to reflect a fascination with or craving of sensory stimulation that is intense or unusual and may be repetitive in nature (e.g., child uses peripheral vision to sight objects, repeatedly rubs a texture, or sniffs objects) [17, 21–24]. These sensory response patterns are evident across sensory modalities, and they often coexist within individuals with ASD [12, 23].

 No studies found in the literature specifically address the potential for sensory response patterns to be a factor constraining the development of verbal communication (i.e., by grouping participants based on the presence or absence of verbal communication) in children with ASD. That is, although the impact of sensory processing problems on verbal communication has been hypothesized (e.g., [9, 25]), previous studies have not examined this question by grouping participants based on the presence or absence of verbal communication. Sensory responsiveness and communication may be associated in children from very early infant development. Both prospective and retrospective studies have shown that social-communication impairments and sensory-motor abnormalities coexist in infants who later developed ASD [26, 27]. Early in development, poor orienting to a communication partner may be a manifestation of unusual sensory processing [27]. A finding that has been replicated in the literature is that infants with ASD exhibit decreased orienting to social stimuli (e.g., [27, 28]). An inability to adequately orient to a communication partner and engage in dyadic attention precludes or at least hinders participation in further social and communicative interaction. Clifford and Dissanayake [29] suggest that dyadic attention, also impaired in ASD, requires both orienting and sustaining attention to social stimuli and plays a role in the development of joint attention. Joint attention (i.e., shared attention between two communication partners and an object or third person) is a robust predictor of language development in typically developing children as well as in children with ASD, with impairments frequently found in children with ASD [30–33]. Poor sensory responsiveness exhibited through decreased orienting is potentially the first observable measure of hyporesponsiveness that negatively impacts joint attention and later development of language [25, 34]. 

In preschool samples, empirical evidence indicates that hyporesponsiveness to environmental stimuli, including social stimuli, is concurrently associated with poor communication in children with ASD. Using cluster analysis, Liss et al. [23] found one cluster of individuals with ASD characterized by high levels of hyporesponsiveness and sensory seeking behaviors along with poor communication. Hilton et al. [16] found that three sensory response patterns (hyporesponsiveness, hyperresponsiveness, and sensory seeking) were associated with social-communicative symptom severity. Similarly, Watson et al. [9] found that hyporesponsiveness and sensory seeking, but not hyperresponsiveness, were negatively correlated with language and social-communication adaptive skills and positively correlated with social-communication symptoms of autism. In partial contrast, Lane et al. [35] used a stepwise multiple regression analysis with 54 children with ASD and found that sensory response patterns explained 24.5% of the variance in communication scores. This finding was largely accounted for by two of the seven subscales on the short sensory profile McIntosh et al., [36] predicting in opposite directions—specifically, “underresponsive/seeks sensation” was associated with lower communication adaptive skills, whereas “low energy/weak” was associated with higher communication adaptive skills.

In sum, unusual sensory features are highly prevalent and often observable at very young ages in children with ASD, and, in several studies by different research groups, sensory response patterns have been found to be related to communication in children with ASD.

Due to the substantial portion of children with autism who remain nonverbal and the associated poor prognoses for their long-term outcomes, research directed at understanding why some children with autism develop functional verbal communication and others do not is a current national priority [8]. With some inconsistencies in results, previous research has provided evidence of associations between communication and sensory response patterns, especially hyporesponsiveness and sensory seeking. The need to understand early behavioral features that may manifest in lack of spoken language by five years of age is of particular importance, due to indications that any spoken language by that age is predictive of eventual development of functional verbal communication. Aiming to examine the extent to which sensory response patterns are associated with the verbal status of young children with ASD, our research questions were as follows: (1) to what extent do sensory response patterns differ between verbal and nonverbal children with ASD and (2) to what extent are early sensory response patterns related to later verbal status in young children with ASD? Based on previous research, we hypothesized that two sensory response patterns, sensory hyporesponsiveness and sensory seeking, would be concurrently and predictively associated with verbal status (i.e., verbal versus nonverbal).

## 2. Method

We examined two extant datasets to address our research questions. The first study (Study 1) used data from all children with ASD who had been originally recruited as part of a larger study examining the development, functional impact, and cause of various sensory features in children with ASD, developmental delay, and/or typical development. The second dataset from which we derived Study 2 involved a subset of children from Study 1 of young, initially low verbal or nonverbal children; we included all participants from the second dataset for our Study 2 analyses. Study 1 included substantially more participants with data available for cross-sectional analyses; Study 2 participants were evaluated at two different time points and thus provided data for longitudinal analyses. 

### 2.1. Participants

 Children were recruited into Study 1 from a university-based research subject registry, developmental clinics, other research groups, parent support groups, local public schools, and the project website. A total of 79 children with a diagnosis of ASD (74 with autism, 5 with broader autism spectrum disorder) ranging from 25 to 89 months (M = 53; SD = 16.57) were included in this study. The participants with ASD largely overlapped with those in another study [9]. See Table 1 for participant and family descriptive and demographic information. The families received a monetary incentive ($75) for participation in the study and children received a small toy or book. The university's institutional review board approved the research, and a parent provided an informed consent for their child's participation. Participants had a diagnosis of ASD confirmed by the research staff through both the *Autism Diagnostic Interview—Revised* (ADI-R [37]) and *Autism Diagnostic Observation Schedule* (ADOS [38]). Participants were also required to have normal or corrected-to-normal hearing and vision as confirmed by screenings performed prior to assessment. Children were excluded if they presented with MA < 6 months, cerebral palsy, seizure disorder, or genetic conditions associated with a high risk of autism (e.g., fragile X, tuberous sclerosis), or were currently receiving psychopharmacological treatments as indicated by parents or from medical records.

ADOS Module 1, item A.1, was used for Study 1 to assign participants to either the verbal or nonverbal group, as in previous research [39], because it specifically quantifies the level of nonechoed language. Nonverbal status was assigned if the participant received a score of three (at least one word or word approximation but fewer than five words used during the session) or a score of eight (no words or word approximations). A child was considered verbal if she/he received a score of 0, 1, or 2 indicating at least 5 single, meaningful, and nonimitative words during the session. ADOS Module 1 is reserved for children who are preverbal or only produce single words. Some children in the verbal group received ADOS Module 2, used with children who use some multiword phrases but are not verbally fluent (i.e., expressive language level below that of a typical four years old); however, the majority of children from the Study 1 dataset (*n* = 51) received Module 1.

A subset of 14 children from Study 1, ranging from 28 to 42 months at entry (M = 35.79; SD = 4.34), were seen longitudinally in Study 2. Inclusion in Study 2 was restricted to children defined as having low verbal ability (i.e., no multiword utterances) at study entrance as determined by ADOS Module 1, item A.1 (overall level of nonechoed language). At the second time point, eight months later, 6 children were classified as verbal while 8 children maintained a nonverbal status based on ADOS results. For Study 2, parents received $25 and the children received a small toy or book for their contributions both for the entry assessments and the follow-up assessments.

### 2.2. Measures

#### 2.2.1. Nonverbal Cognitive Measures

An estimate of nonverbal mental age was obtained from either the *Mullen Scales of Early Learning *(MSEL [40]) or the Stanford-Binet Intelligence Scales Fifth Edition (SB-5 [41]), depending on the child's age/ability level; 76 participants received the MSEL, and three participants received the SB-5. The MSEL is a standardized, examiner-administered measure of cognitive functioning for children from birth to 68 months of age. The SB-5 is a standardized, examiner-administered measure of cognitive functioning for individuals of ages 2 to 85 years. Nonverbal mental ages were used to avoid conflation with verbal ability. An IQ proxy was computed by dividing the nonverbal mental age from each assessment by the child's chronological age and multiplying by 100, as an estimate of cognitive functioning. We computed these IQ proxy scores due to the large number of children who scored at the lowest possible standard score on the MSEL or SB-5; thus, using the standard scores would have introduced floor effects and constrained variability in the analysis. 

#### 2.2.2. Sensory Measures

The *Sensory Experiences Questionnaire, version 2.1* (SEQ 2.1 [11, 42, 43]) is a 42-item caregiver questionnaire that assesses sensory responsiveness (hyporesponsiveness, hyperresponsiveness, and sensory seeking) across modalities (e.g., visual, tactile, and auditory) for children of ages from 6 months to 12 years. The SEQ has good psychometric properties: internal consistency (*α* = .800) and test-retest reliability (ICC = .92) [43]. The *Sensory Profile* (SP [44, 45]) is a commonly used 125-item caregiver questionnaire for children of aged from 3 to 10 designed to assess sensory processing across modalities in a variety of clinical populations including children with autism [45]. The *Sensory Processing Assessment for Young Children* (SPA [25, 46, 47]) is a 20-minute clinically administered observational measure assessing sensory processing across modalities for children ages 9 months to 6 years. This protocol is semistructured and play based, providing behavioral presses to elicit various sensory responses in the tactile, auditory, and visual modalities. Intraclass correlation coefficients ranged from .91 to .99, indicating good reliability for scales. The *Tactile Defensiveness and Discrimination Test-Revised* (TDDT-R [48, 49]) is clinically administered and assesses tactile processing, specifically hyperresponsiveness and discrimination in preschool and school-age children. Presses are in the tactile modality and include touch stimuli applied to the participant's skin (e.g., finger puppet/cotton swab; sticker) and exploration of various tactile materials (e.g., sand, lotion, vibration, carpet, dried noodles, etc.).

A multitrait, multimethod approach (in this case, both parent report and observational methods) has been recommended in the measurement of psychological constructs [50, 51]. Consistent with such recommendations, this study utilized a factor analytic model created from these four sensory processing assessments, yielding standardized factor scores for each participant for each of the three sensory construct scores: hyperresponsiveness (HYPER), hyporesponsiveness (HYPO), and sensory seeking (SEEK). We created a measurement model in MPLUS (version 5) that had 5 latent constructs (observational measures, parent measures, HYPO, HYPER, and SEEK) and 11 manifest variables (each sensory pattern represented in each of the four sensory assessments; the TDDT-R only contributed HYPER and SEEK items, whereas the SEQ, SP, and SPA each contributed HYPO, HYPER, and SEEK items). The factor analytic model allowed for correction of rater effect (error variance) attributable to parent reporting in two instruments versus observational measurement in two other instruments. The measurement model had good fit, as shown by a nonsignificant chi-square, *χ* ^2^ (1, *N* = 110) = 32.4, *P* = .40; a comparative fit index (CFI) near 1 (CFI = 0.997); and a root-mean-square error of approximation (RMSEA) <.08 (RMSEA = .021).

## 3. Results

 Results from chi-square analyses and *t*-tests revealed several demographic characteristics that differed between the verbal and nonverbal groups in the Study 1 cross-sectional data. Verbal children were more likely to be white and older, have higher IQ proxy scores, have mothers with higher education, and have families with higher incomes. Verbal status was not associated with gender or ethnicity. See Table 1 for results. Demographic analyses (sex, ethnicity, race, chronological age, IQ, maternal education, household income) for our Study 2 longitudinal data did not show statistically significant differences between children who were verbal versus children who remained nonverbalwere not, which is not surprising given the small sample size. Additionally, our criteria that children who entered Study 2 have low verbal status may have resulted in more homogeneity within the sample. Language measures were obtained for children in Study 2 (longitudinal), and children who became verbal at the second time point had better receptive and expressive language at the first time point, including more gestures, than children who remained nonverbal. Thus, although all children met the criteria for low verbal status at study entry, differences in communication abilities were already evident. See Table 2 for results. The number of hours of participation in speech language therapy between study entry and eight months later was compared between groups and yielded no significant differences (*P* = .61). 

**Figure 1:**
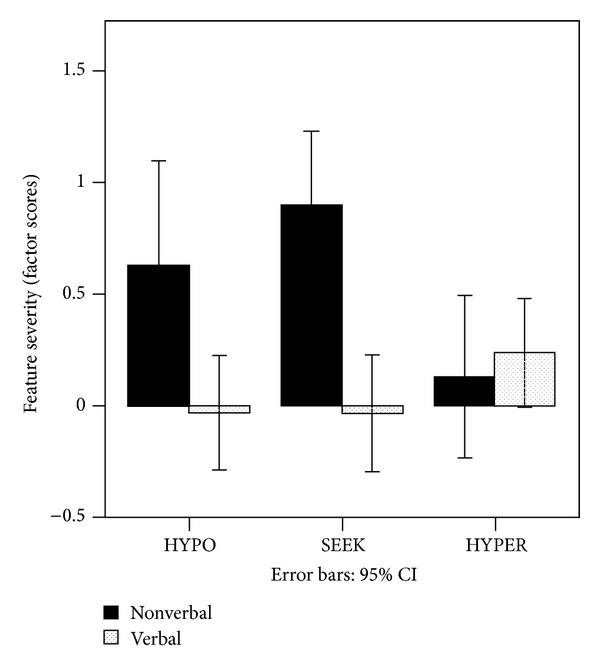
Level of severity of sensory response patterns for verbal and nonverbal subgroups in the cross-sectional sample.

*Research Question  1: To What Extent Do Sensory Response Patterns Differ between Verbal and Nonverbal Children with ASD?* Sensory patterns of verbal and nonverbal children from Study 1 (cross-sectional) were contrasted using a mixed ANOVA. The within subjects variable was the type of sensory response pattern (HYPO, SEEK, and HYPER) and the between subjects variable was verbal status (verbal versus nonverbal). This design allowed us to examine differences in performance across the three sensory response patterns, overall differences between verbal status across sensory patterns, and the sensory pattern versus verbal status interaction. The analyses were conducted with SPSS (PASW Statistics 18). Means are presented in Figure 1. The mixed ANOVA results indicated that there were nosignificant differences across the three sensory patterns, *F*(2,77) = 1.05, *P* = .31. However, there was a significant main effect for verbal status, *F*(1,77) = 10.59, *P* = .002, and a significant verbal status by sensory response pattern interaction; *F*(2,77) = 7.59, *P* = .001. The nature of this interaction was examined using planned interaction contrasts [52]. These results indicate that the nonverbal group was significantly higher than the verbal group on HYPO (*P* = .008) and SEEK (*P* < .001) but not significantly different on HYPER, *P* = .61. Effect sizes for both HYPO and SEEK were large based on Cohen's *d* (Cohen, 1988), HYPO (*d* = .56); SEEK (*d* = .62). The effect size for HYPER was small (*d* = .11). 

**Figure 2:**
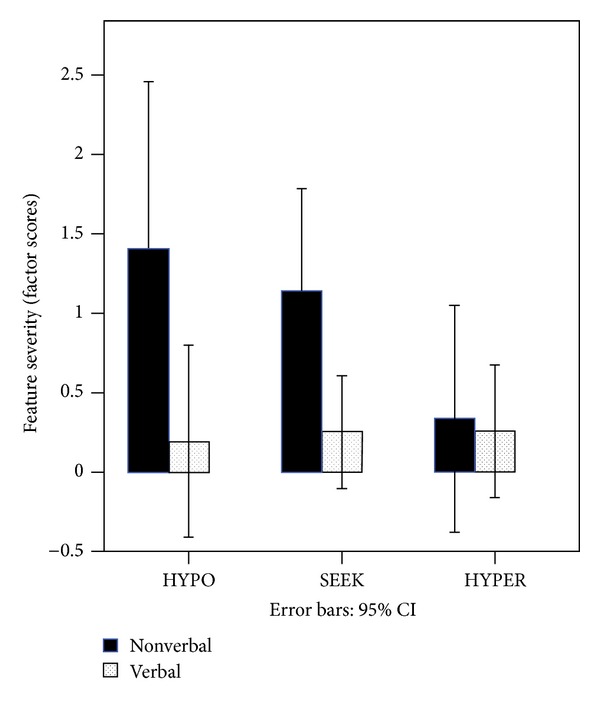
Level of severity of sensory response patterns for verbal and nonverbal subgroups in the longitudinal sample.

*Research Question  2: To What Extent Are Early Sensory Features Related to Later Verbal Status in Preschool Children with ASD?* Using the same analyses as above, we examined longitudinal data for 14 children from our Study 2 dataset who each met the criteria for low verbal status at study entry. The children were assigned to either the verbal or the nonverbal group based upon the ADOS assessment administered one year after the sensory measures were taken. Means for sensory scores are presented in Figure 2. The mixed ANOVA results indicated no significant differences across the three sensory response patterns, *F*(2,12) = .09, *P* = .77. There was a significant main effect for verbal status across the three sensory response patterns, *F*(1,12) = 7.43, *P* = .018; however, there was no significant verbal status by sensory response pattern interaction, *F*(2,24) = 1.94, *P* = .17. Pairwise comparisons were used to examine group differences. These results indicate that the nonverbal group was significantly higher than the verbal group on HYPO (*P* = .05) and SEEK (*P* = .02) but not significantly different on HYPER, *P* = .84. Effect sizes were calculated using Hedge's *g* to account for small sample sizes and were large for both HYPO and SEEK: HYPO (*g* = 1.47), SEEK (*g* = 1.16). The effect size for HYPER was small (*g* = .12).

## 4. Discussion

 We examined associations between sensory response patterns and verbal status in children with ASD. Our cross-sectional and longitudinal data demonstrated that higher levels of sensory hyporesponsiveness and sensory seeking were both concurrently and predictively associated with nonverbal status, as we initially predicted. Sensory hyperresponsiveness did not differentiate children who were verbal from children who were nonverbal in either the cross-sectional or the longitudinal data. Our findings support those of other research teams indicating that both sensory hyporesponsiveness and sensory seeking behaviors are inversely associated with language development [9, 16, 23, 34]. Also, the current studies confirm and elaborate on our previously reported findings on many of these same participants [9, 25] by explicitly examining the association of sensory response patterns with verbal versus nonverbal status and demonstrating for the first time that hyporesponsiveness and sensory seeking patterns also predict longitudinally later verbal or nonverbal status. Both hyporesponsiveness and sensory seeking patterns may reflect established deficits in attention orienting and shifting in children with ASD (e.g., [34, 53, 54]) that may constrain communication development. Measures of hyporesponsiveness are not clearly separable from attention orienting, in that failure to look toward the source of a novel sound can be labeled as both poor attention orienting as well as hyporesponsiveness to sensory stimuli. Attention orienting is a necessary gateway to the larger construct of attention that involves sustaining and shifting attention and allows for deeper processing of information. Likewise, sensory seeking behaviors that involve an intense focus on stimuli preferred by the child may preclude attention orienting to other important stimuli (e.g., conventional toys and/or stimuli being referenced in language directed to the child) and in essence have the same result as hyporesponsiveness. It may be that unusual sensory responses result in some of the communication symptoms associated with autism, but a separate underlying factor may result in both unusual sensory responses and communication symptoms; thus the two may not be causally related.

Neural deficits in early stages of sensory processing may result in behavioral patterns of sensory hyporesponsiveness and contribute to inadequate attention to environmental stimuli, thus hindering the ability to process and learn from both ambient social-communication as well as direct bids for interaction. Early in development, caregiver-generated stimuli are laden with social and communicative elements. Typically developing infants are drawn to such dyadic interactions, but infants with ASD demonstrate impairments in dyadic attention [29]. Thus, early sensory processing deficits could contribute to the lack of attention to dyadic interactions as well as other environmental stimuli. Sensory seeking behavior (e.g., fixation on light patterns) also may constrain social and communication learning opportunities due to over-focused attention to nonsocial stimuli. Thus, both of these sensory response patterns may adversely impact a child's ability to take advantage of important social and communicative learning opportunities. 

 Our findings regarding demographic differences between verbal and nonverbal children indicate that verbal children come from families who are white, with higher education and income. Although this is a sample of convenience rather than an epidemiological sample, these findings may reflect disparities in services that are related to demographic variables. For example, in an analysis of service utilization, in which the sample largely overlapped that in the current study, higher parental education was related to more intervention services obtained [55]. 

### 4.1. Clinical Implications

 This study adds to the growing body of research linking sensory hyporesponsiveness and sensory seeking patterns to poorer communication outcomes in children with ASD [9, 16, 23]. Because sensory hyporesponsiveness may be observable in infancy, these findings have implications for early detection and intervention and suggest that children with autism may benefit from techniques to enhance capture of attention to salient social and communicative stimuli perhaps through enhancements of perceptual elements and/or motivational systems. Many available clinical interventions incorporate techniques to enhance attention (e.g., audio-visual pairings such as a pointing gesture with a verbal command, “look” [56]). For a review of additional attention enhancing techniques, see [57]. Therapeutic interventions to enhance sensory processing functions are popular in the treatment of ASD [58], but the efficacy of these treatments is mixed and debated among researchers [59–61]; thus more research is needed particularly with respect to communication outcomes. 

### 4.2. Limitations and Future Direction

This study was based on two extant databases that were not specifically designed to address the impact of sensory features on verbal status. Our longitudinal dataset was small (*n* = 14) but provided unique information regarding the predictive nature of early sensory features. Future studies should examine trajectories of attainment of verbal status, at a variety of age ranges, as a function of sensory features both concurrently and predictively in larger samples. 

Our assessments were all behavioral measures relying on observation or parent report. Sensory processing and language development may share some neurobiological mechanisms that require bio-physiological measures to adequately assess them. Therefore, future studies should include more direct measures of potential underlying neurological mechanisms. 

 Our analyses revealed significant demographic differences between verbal and nonverbal children with autism. Future studies should explore the stability of these findings and why the differences exist. Further, factors such as access to services should be included as potential mediators between demographic differences (e.g., income disparity) and developmental outcomes. 

## Figures and Tables

**Table 1:** Demographic information for cross-sectional dataset.

	Nonverbal (*N* = 29)	Verbal (*N* = 50)	Total (*N* = 79)
Male	24 (30%)	43 (54%)	67 (85%)
Hispanic	1 (1%)	3 (4%)	4 (5%)
race^∗,+^			
Asian	1 (3%)	0 (0%)	1 (1%)
Black	7 (24%)	2 (4%)	9 (11%)
White	16 (55%)	46 (92%)	62 (79%)
Multiple races	5 (17%)	2 (4%)	7 (9%)
Chronological age^∗∗,+^	43.5 (12.74) mos	57.9 (16.35) mos	
IQ (proxy)^∗∗∗,+^	33.8 (12.38)	73.5 (25.28)	
Maternal education^∗∗∗∗,++^			
Partial high school	0 (0%)	1 (2%)	1 (1%)
High school/GED	8 (28%)	7 (14%)	15 (19%)
Associate/partial college	11 (38%)	8 (16%)	19 (24%)
Bachelor	8 (28%)	22 (44%)	30 (38%)
Masters/doctorate	2 (7%)	12 (24%)	14 (18%)
Household income^∗∗∗∗∗,+^			
<$20000	0 (0%)	1 (2%)	1 (1%)
$20000–59999	19 (66%)	15 (30%)	34 (43%)
$60000–99999	9 (31%)	20 (40%)	29 (37%)
>$100000	1 (3%)	14 (28%)	15 (19%)

^+^Significant differences based on verbal/nonverbal status at *P* < .01.

^++^Significant differences based on verbal/nonverbal status at *P* < .05.

*Verbal children were more likely to be white *X* ^2^(3, *N* = 79) = 15.06, *P* = .002.

**Verbal children were older *t*(77) = −4.56, *P* < .001.

***Verbal children had higher IQs *t*(77) = −7.90, *P* < .001.

****Mothers of verbal children tended to have more education *X* ^2^(4, *N* = 79) = 10.37, *P* = .04.

*****Verbal children tended to come from households with higher income *X* ^2^(3, *N* = 79) = 12.19, *P* = .007.

**Table 2:** Language assessment means and standard deviations for the longitudinal sample at study initiation.

Language assessment	Status at T2 (nonverbal *n* = 8) (verbal *n* = 6)	Mean	SD
PLS total AE*	Nonverbal	7.25	3.11
	Verbal	16.33	5.85
PLS AC, AE	Nonverbal	6.25	5.12
	Verbal	16.50	7.48
PLS EC, AE*	Nonverbal	9.38	2.97
	Verbal	18.00	6.13
MCDI, EG	Nonverbal	7.71	3.60
	Verbal	11.50	4.09
MCDI, LG*	Nonverbal	9.71	7.04
	Verbal	22.50	2.74
Number of gestures used*	Nonverbal	17.43	10.26
	Verbal	34.00	6.20

*P < .05.

PLS: Preschool Language Scale, version 4.

AE: age equivalent (AE used due to floor effects with standard scores).

AC: auditory comprehension.

EC: expressive communication.

MCDI: MacArthur-Bates Communicative Development Inventories.

EG: early gestures.

LG: late gestures.

## References

[1] Baghdadli A, Assouline B, Sonié S (2012). Developmental trajectories of adaptive behaviors from early childhood to adolescence in a cohort of 152 children with autism spectrum disorders. *Journal of Autism and Developmental Disorders*.

[2] Lord C, Risi S, Pickles A, Rice ML, Warren SF (2004). Trajectory of language development in autistic spectrum disorders. *Developmental Language Disorders: From Phenotypes to Etiologies*.

[3] Sigman M, McGovern CW (2005). Improvement in cognitive and language skills from preschool to adolescence in autism. *Journal of Autism and Developmental Disorders*.

[4] Pickett E, Pullara O, O’Grady J, Gordon B (2009). Speech acquisition in older nonverbal individuals with autism: a review of features, methods, and prognosis. *Cognitive and Behavioral Neurology*.

[5] Kobayashi R, Murata T, Yoshinaga K (1992). A follow-up study of 201 children with autism in Kyushu and Yamaguchi areas, Japan. *Journal of Autism and Developmental Disorders*.

[6] Venter A, Lord C, Schopler E (1992). A follow-up study of high-functioning autistic children. *Journal of Child Psychology and Psychiatry and Allied Disciplines*.

[7] Tager-Flusberg H, Rogers S, Cooper J (2009). Defining spoken language benchmarks and selecting measures of expressive language development for young children with autism spectrum disorders. *Journal of Speech, Language, and Hearing Research*.

[8] Gilotty L Meeting summary. *NIH workshop on nonverbal school-aged children with autism*. http://www.nimh.nih.gov/research-funding/scientific-meetings/2010/nih-workshop-on-nonverbal-school-aged-children-with-autism/nih-workshop-on-nonverbal-school-aged-children-with-autism.shtml.

[9] Watson LR, Patten E, Baranek GT (2011). Differential associations between sensory response patterns and language, social, and communication measures in children with autism or other developmental disabilities. *Journal of Speech, Language, and Hearing Research*.

[10] Ben-Sasson A, Hen L, Fluss R, Cermak SA, Engel-Yeger B, Gal E (2009). A meta-analysis of sensory modulation symptoms in individuals with autism spectrum disorders. *Journal of Autism and Developmental Disorders*.

[11] Cesaroni L, Garber M (1991). Exploring the experience of autism through firsthand accounts. *Journal of Autism and Developmental Disorders*.

[12] Baranek GT, David FJ, Poe MD, Stone WL, Watson LR (2006). Sensory experiences questionnaire: discriminating sensory features in young children with autism, developmental delays, and typical development. *Journal of Child Psychology and Psychiatry and Allied Disciplines*.

[13] Lord C, Rutter M, Couteur AL (1994). Autism diagnostic interview-revised: a revised version of a diagnostic interview for caregivers of individuals with possible pervasive developmental disorders. *Journal of Autism and Developmental Disorders*.

[14] Rogers SJ, Hepburn S, Wehner E (2003). Parent reports of sensory symptoms in toddlers with autism and those with other developmental disorders. *Journal of Autism and Developmental Disorders*.

[15] Wiggins LD, Robins DL, Bakeman R, Adamson LB (2009). Breif report: sensory abnormalities as distinguishing symptoms of autism spectrum disorders in young children. *Journal of Autism and Developmental Disorders*.

[16] Hilton C, Graver K, LaVesser P (2007). Relationship between social competence and sensory processing in children with high functioning autism spectrum disorders. *Research in Autism Spectrum Disorders*.

[17] Boyd BA, Baranek GT, Sideris J (2010). Sensory features and repetitive behaviors in children with autism and developmental delays. *Autism Research*.

[18] Foss-Feig JH, Heacock JL, Cascio CJ (2012). Tactile responsiveness patterns and their association with core features in autism spectrum disorders. *Research in Autism Spectrum Disorders*.

[19] Neumann N, Dubischar-Krivec AM, Poustka F, Birbaumer N, Bölte S, Braun C (2011). Electromagnetic evidence of altered visual processing in autism. *Neuropsychologia*.

[20] O’Connor K (2012). Auditory processing in autism spectrum disorder: a review. *Neuroscience and Biobehavioral Reviews*.

[21] Ashburner J, Ziviani J, Rodger S (2008). Sensory processing and classroom emotional, behavioral, and educational outcomes in children with autism spectrum disorder. *American Journal of Occupational Therapy*.

[22] Ben-Sasson A, Cermak SA, Orsmond GI, Tager-Flusberg H, Kadlec MB, Carter AS (2008). Sensory clusters of toddlers with autism spectrum disorders: differences in affective symptoms. *Journal of Child Psychology and Psychiatry and Allied Disciplines*.

[23] Liss M, Saulnier C, Fein D, Kinsbourne M (2006). Sensory and attention abnormalities in autistic spectrum disorders. *Autism*.

[24] Tomchek SD, Dunn W (2007). Sensory processing in children with and without autism: a comparative study using the short sensory profile. *American Journal of Occupational Therapy*.

[25] Baranek GT, Watson LR, Boyd BA, Poe MD, David FJ, Mcguire L (2013). Hyporesponsiveness to social and nonsocial sensory stimuli in children with autism, children with developmental delays, and typically developing children. *Development and Psychopathology*.

[26] Bryson SE, Zwaigenbaum L, Brian J (2007). A prospective case series of high-risk infants who developed autism. *Journal of Autism and Developmental Disorders*.

[27] Baranek GT (1999). Autism during infancy: a retrospective video analysis of sensory-motor and social behaviors at 9–12 months of age. *Journal of Autism and Developmental Disorders*.

[28] Werner E, Dawson G, Osterling J, Dinno N (2000). Brief report: recognition of autism spectrum disorder before one year of age: a retrospective study based on home videotapes. *Journal of Autism and Developmental Disorders*.

[29] Clifford SM, Dissanayake C (2008). The early development of joint attention in infants with autistic disorder using home video observations and parental interview. *Journal of Autism and Developmental Disorders*.

[30] Carpenter M, Nagell K, Tomasello M (1998). Social cognition, joint attention, and communicative competence from 9 to 15 months of age. *Monographs of the Society for Research in Child Development*.

[31] Charman T, Swettenham J, Baron-Cohen S, Cox A, Baird G, Drew A (1997). Infants with autism: an investigation of empathy, pretend play, joint attention, and imitation. *Developmental psychology*.

[32] Leekam SR, López B, Moore C (2000). Attention and joint attention in preschool children with autism. *Developmental Psychology*.

[33] McArthur D, Adamson LB (1996). Joint attention in preverbal children: autism and developmental language disorder. *Journal of Autism and Developmental Disorders*.

[34] Dawson G, Toth K, Abbott R (2004). Early social attention impairments in autism: social orienting, joint attention, and attention to distress. *Developmental Psychology*.

[35] Lane AE, Young RL, Baker AEZ, Angley MT (2010). Sensory processing subtypes in autism: association with adaptive behavior. *Journal of Autism and Developmental Disorders*.

[36] McIntosh DN, Miller LJ, Shyu V, Dunn W, Dunn W (1999). Overview of the short sensory profile. *The SenSory Profile Examiner's Manual*.

[37] le Couteur A, Lord C, Rutter M (2003). *Autism Diagnostic Interview-Revised (ADI-R)*.

[38] Lord C, Rutter M, Dilavore P, Risi S (1999). *The Autism Diagnostic Observation Schedule (ADOS)*.

[39] Gordon K, Pasco G, McElduff F, Wade A, Howlin P, Charman T (2011). A communication-based intervention for nonverbal children with autism: What changes? Who benefits?. *Journal of Consulting and Clinical Psychology*.

[40] Mullen EM (1995). *Scales of Early Learning (AGS Edition)*.

[41] Roid GH (2003). *,Stanford Binet Intelligence Scales*.

[42] Baranek GT (1999). *Sensory Experiences Questionnaire (SEQ)*.

[43] Little LM, Freuler AC, Houser MB (2011). Psychometric validation of the sensory experiences questionnaire. *American Journal of Occupational Therapy*.

[44] Dunn W (1999). *Sensory Profile*.

[45] Kientz MA, Dunn W (1997). A comparison of the performance of children with and without autism on the sensory profile. *American Journal of Occupational Therapy*.

[46] Baranek GT (1999). *Sensory Processing Assessment for Young Children (SPA)*.

[47] Baranek GT, Boyd BA, Poe MD, David FJ, Watson LR (2007). Hyperresponsive sensory patterns in young children with autism, developmental delay, and typical development. *American Journal on Mental Retardation*.

[48] Baranek GT (1998). *Tactile Defensiveness and Discrimination Test: Revised*.

[49] Baranek GT, Berkson G (1994). Tactile defensiveness in children with developmental disabilities: responsiveness and habituation. *Journal of Autism and Developmental Disorders*.

[50] Eid M, Nussbeck FW, Geiser C, Cole DA, Gollwitzer M, Lischetzke T (2008). Structural equation modeling of multitrait-multimethod data: different models for different types of methods. *Psychological Methods*.

[51] John OP, Benet-Martinez V, Reis HT, Judd CM (2000). Measurement: reliability, construct validation, and scale construction. *Handbook of Research Methods in Social and Personality Psychology*.

[52] Keppel G, Zedeck S (1989). *Data Analysis For Research Designs: Analysis of Variance and Multiple Regression/Correlation Approaches*.

[53] Landry R, Bryson SE (2004). Impaired disengagement of attention in young children with austism. *Journal of Child Psychology and Psychiatry and Allied Disciplines*.

[54] Swettenham J, Baron-Cohen S, Cox A (1998). The frequency and distribution of spontaneous attention shifts between social and nonsocial stimuli in autistic, typically developing, and nonautistic developmentally delayed infants. *Journal of Child Psychology and Psychiatry and Allied Disciplines*.

[55] Patten E, Baranek GT, Watson LR, Schultz BS (2012). Child and family characteristics influencing intervention choices in autism spectrum disorders. *Focus on Autism and Other Developmental Disabilities*.

[56] Leekam SR, Hunnisett E, Moore C (1998). Targets and cues: gaze-following in children with autism. *Journal of Child Psychology and Psychiatry and Allied Disciplines*.

[57] Patten E, Watson LR (2011). Interventions targeting attention in young children with autism. *American Journal of Speech-Language Pathology*.

[58] Hodgetts S, Hodgetts W (2007). Somatosensory stimulation interventions for children with autism: Literature review and clinical considerations. *Canadian Journal of Occupational Therapy*.

[59] Baranek GT (2002). Efficacy of sensory and motor interventions for children with autism. *Journal of Autism and Developmental Disorders*.

[60] Lang R, O’Reilly M, Healy O (2012). Sensory integration therapy for autism spectrum disorders: a systematic review. *Research in Autism Spectrum Disorders*.

[61] National Autism Center (2009). *National Standards Report: Addressing the Need for Evidence-Based Practice Guidelines for Autism Spectrum Disorders*.

